# Comparison of Readability Scores for Written Health Information Across Formulas Using Automated vs Manual Measures

**DOI:** 10.1001/jamanetworkopen.2022.46051

**Published:** 2022-12-12

**Authors:** Olivia Mac, Julie Ayre, Katy Bell, Kirsten McCaffery, Danielle M. Muscat

**Affiliations:** 1 The University of Sydney Faculty of Medicine and Health, School of Public Health, Sydney, New South Wales, Australia; 2 Sydney Health Literacy Lab, The University of Sydney Faculty of Medicine and Health, School of Public Health, Sydney, New South Wales, Australia

## Abstract

This cross-sectional study examines the variability of readability scores across widely used online calculators.

## Introduction

Assessing the readability of written health information is a common way to evaluate whether patients are likely to understand it.^1^ Readability is an objective measure that estimates a text’s equivalent school-grade reading level and is increasingly recommended globally in health policies.^2,3^ Several formulas for calculating readability exist, and scores can vary substantially depending on the formula applied.^4^ There has also been a proliferation of automated online calculators that provide readability estimates within seconds. However, the accuracy and consistency of automated calculators have not been evaluated.

The aims of this study were to assess (1) the variability of readability scores across automated calculators, (2) the association of text preparation with score variability, and (3) the level of agreement of automated readability scores with the reference standard (manually calculated scores) using the Simple Measure of Gobbledygook (SMOG) Index, the Flesch Kincaid Grade Level (FKGL), and the Automated Readability Index (ARI).

## Methods

This cross-sectional study followed the STROBE reporting guideline. Ethical approval was not required because all information was in the public domain, and no human participants were involved.

In April 2022, we identified automated readability calculators from the published literature and wide application in Australia (eMethods and eTable in Supplement 1). We selected 2 webpages from 5 health topics linked on the Centers for Disease Control and Prevention website: COVID-19, attention-deficit/hyperactivity disorder, chronic obstructive pulmonary disease, diabetes, and cancer.

Two scores were obtained by each calculator: one using unedited text and one in which the text was prepared based on guidelines for readability assessment (eg, removal of incomplete sentences and midsentence periods) (eMethods in Supplement 1).^5^ We calculated the proportion of text excluded in the preparation process. We reported the SMOG Index, FKGL, and ARI to capture formulas used across all included calculators.

To provide a reference standard for determining the accuracy of automated scores, we calculated the SMOG index, FKGL, and ARI scores manually for the prepared text (eMethods in Supplement 1). Agreement with the reference standard was assessed using Bland-Altman plots.^6^ Comparisons were made across formulas, calculators, and methods of text preparation. A 95% limit of agreement less than 1 grade was considered good agreement; 2 grades or above was considered poor agreement and therefore inaccurate. Data were analyzed using R, version 4.1.2 (R Foundation for Statistical Computing). Bland-Altman plots were constructed using the package ggplot2.

## Results

We identified 8 readability calculators: Microsoft Word, Online Utility, Readable, Readability Studio, Readability Formula, WebFX, Hemingway App, and the Sydney Health Literacy Lab (SHeLL) Health Literacy Editor. There were 16 combinations of calculator and formula (4 for the SMOG Index, 6 for FKGL, and 6 for ARI).

Across all calculators, the same text produced scores that varied by up to 12.9 grade reading levels even when using the same formula (Table). For all but 3 calculations, text preparation decreased variability among calculators (range, 2.1 grade levels) (Table). However, for 5 of 10 texts, this preparation involved omitting more than 20% of the text (range, 4%-25%).

**Table.** Readability Scores Obtained by Each Formula and Calculator^a^

Text	SMOG Index					FKGL							ARI						
	Ref	Online Utility	Readable	Readability Studio	SHeLL Editor	Ref	Microsoft Word	Online Utility	Readable	Readability Formula	Readability Studio	WebFX	Ref	Hemingway	Online Utility	Readable	Readability Formula	Readability Studio	WebFX
ADHD																			
Unedited	12.9	11.8	11.2	12.8	12.8	10.8	11.0	10.1	8.9	10.1	11.2	10.1	10.8	9.0	9.8	8.6	10.1	12.4	9.8
Prepared		11.3	12.9	11.9	12.8		9.6	9.6	8.7	9.3	9.8	9.3		10.0	9.6	9.9	9.6	10.4	9.6
ADHD treatment																			
Unedited	13.0	13.0	11.6	12.6	12.9	10.2	9.4	11.5	8.5	10.4	10.8	10.8	10.8	9.0	11.2	9.2	11.7	12.1	11.4
Prepared		11.9	12.0	12.1	13.0		9.3	9.8	9.2	9.7	9.2	9.3		10.0	10.0	10.1	10.1	10.4	9.9
Diabetes																			
Unedited	11.9	11.9	11.3	12.3	11.0	9.5	9.0	9.8	7.9	9.6	8.8	8.8	9.2	7.0	8.1	7.5	8.2	9.7	8.0
Prepared		11.5	11.8	11.9	11.8		8.8	9.5	8.7	9.1	8.5	8.5		8.0	7.9	8.5	7.9	8.9	7.9
Diabetes risk factors																			
Unedited	14.1	19.4	12.8	14.5	13.5	11.9	11.7	20.4	9.9	19.6	13.9	19.6	11.2	7.0	19.9	8.1	19.4	14.2	19.4
Prepared		13.8	14.1	13.9	14.4		11.3	12.2	11.2	11.0	11.2	11.0		10.0	10.2	10.7	10.2	11.0	10.2
COPD																			
Unedited	12.7	11.2	11.4	11.7	12.7	10.8	11.3	7.7	8.1	7.3	9.6	7.2	9.3	7.0	6.4	7.5	5.4	10.9	5.0
Prepared		10.0	10.6	11.8	12.9		8.9	8.9	8.6	8.4	9.2	8.4		8.0	8.6	8.5	8.4	8.9	8.4
COPD diagnosis																			
Unedited	12.2	11.1	11.1	11.1	12.3	9.9	7.9	9.0	8.0	8.3	8.2	8.3	8.7	8.0	8.2	7.8	8.3	9.1	8.1
Prepared		10.8	11.2	11.1	12.2		8.1	8.8	8.3	8.1	8.2	8.1		8.0	8.0	8.1	7.9	9.1	7.9
COVID-19 vaccines																			
Unedited	13.7	12.8	11.8	14.5	13.4	12.9	12.2	12.6	9.9	11.6	13.7	11.6	13.0	11.0	12.3	10.3	11.9	14.6	11.7
Prepared		12.4	12.7	13.9	13.8		11.5	11.8	11.1	12.8	10.7	10.7		12.0	11.3	11.8	10.7	12.8	10.7
COVID-19 self-test																			
Unedited	12.3	11.5	10.3	12.6	11.4	9.0	8.7	10.1	7.0	9.1	10.4	9.1	9.2	6.0	8.5	6.0	8.1	13.5	8.1
Prepared		11.1	11.6	12.0	12.2		9.0	9.5	8.6	9.3	8.4	8.5		8.0	8.0	8.2	7.8	9.4	7.8
Healthy choices																			
Unedited	12.6	12.2	11.9	13.9	12.6	11.5	10.9	10.8	10.0	10.0	12.9	10.0	11.8	9.0	9.8	9.2	9.5	12.9	9.3
Prepared		12.4	12.2	12.9	12.5		11.1	11.3	11.0	10.4	12.0	10.4		11.0	10.9	11.0	10.6	11.9	10.6
Breast cancer risk factors																			
Unedited	11.1	10.2	10.4	10.8	10.6	9.6	8.5	8.8	8.2	8.9	8.1	8.1	10.8	9.0	8.6	8.6	8.7	10.1	8.5
Prepared		10.2	10.6	11.0	10.6		9.3	9.7	9.2	8.9	9.8	9.0		10.0	10.1	10.2	10.0	10.6	10.0

Abbreviations: ADHD, attention-deficit/hyperactivity disorder; ARI, Automated Readability Index; COPD, chronic obstructive pulmonary disease; FKGL, Flesch Kincaid Grade Level; ref, reference standard; SHeLL Editor, Sydney Health Literacy Editor; SMOG, Simple Measure of Gobbledygook.

^a^ SHeLL Editor, Hemingway, and Microsoft Word only used a single formula (SMOG, FKGL, and ARI, respectively). SMOG is not reported for Readability Formula or WebFX because these calculators did not apply the formula correctly during the screening process (eMethods in Supplement 1).

Bland-Altman plots for SMOG Index scores are displayed in the Figure. The SMOG Index scores from Readability Studio and SHeLL Editor and the FKGL scores from Microsoft Word showed good agreement with the reference standard. All other calculators showed poor agreement. For example, the 95% limits of agreement for ARI scores from WebFx were 7.1 grades below to 6.0 grades above the reference standard (reduced to 0.3-2.1 grades below the reference standard after text preparation).

**Figure.**
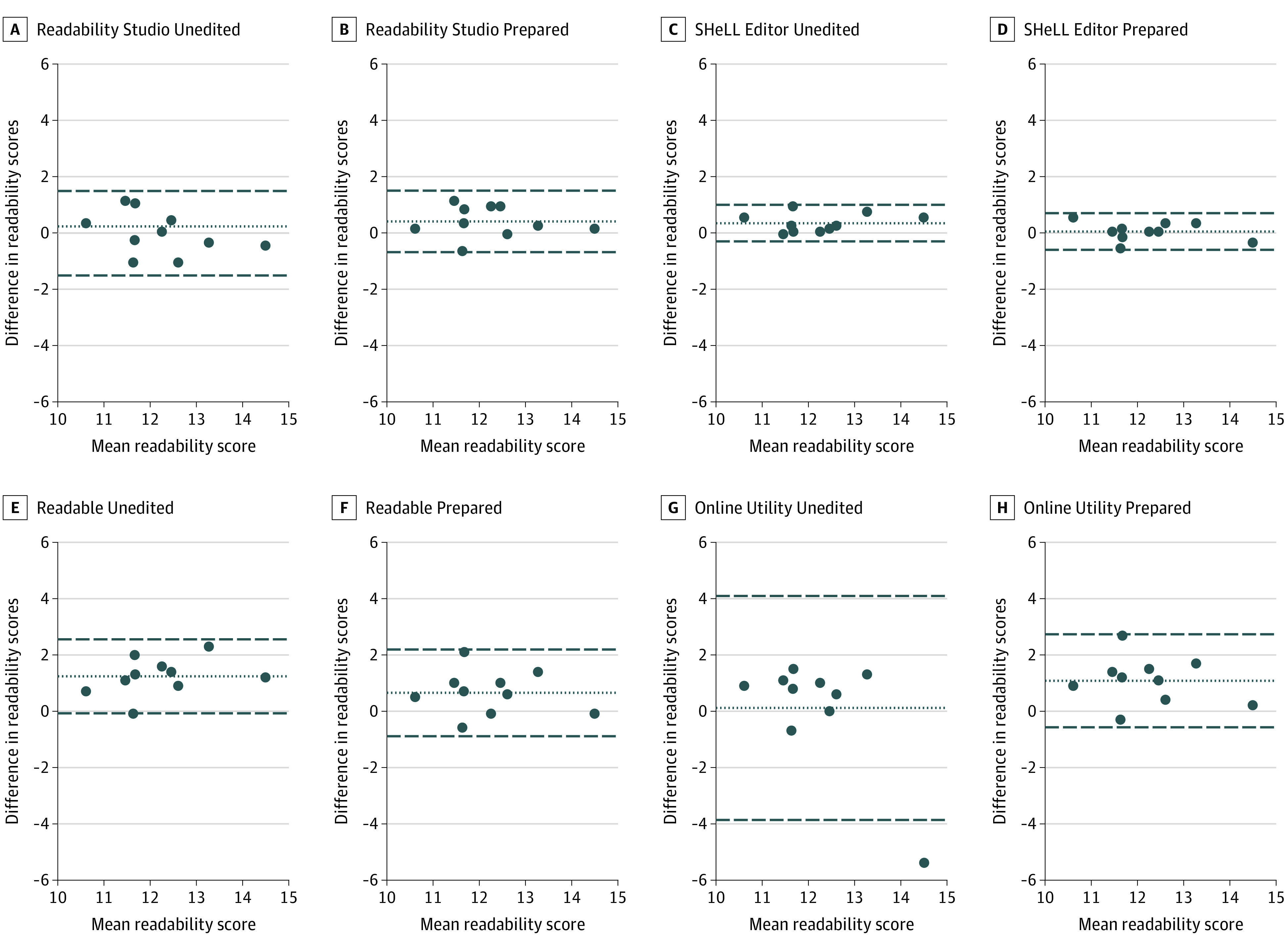
Level of Agreement of Simple Measure of Gobbledygook (SMOG) Index Scores From Online Calculators With the Reference Standard Bland-Altman plots illustrating the agreement of SMOG Index readability scores from online calculators with the reference standard (hand-calculated scores) for the unedited and prepared versions of text. The center line represents the bias or the mean difference in readability scores. The outer lines represent the 95% limits of agreement (mean difference ±1.96 × SD). The closer the mean difference is to zero and the narrower the limits of agreement, the stronger the agreement with the reference standard.

## Discussion

Our findings suggest that automated readability scores are inconsistent and often inaccurate, meaning that, despite good intentions, health information that is revised to meet health literacy guidelines may still be too complex for people to understand. A limitation of this study is that although a difference of 2 reading grade levels from the reference standard is large (our definition of poor agreement), a minimally important difference has not been defined. Comprehensive guidance on the conduct and reporting of readability assessments is needed to improve accuracy of readability scores and the accessibility of written information for patients.

## Notes

Supplement 1. **eMethods.** Detailed Methods **eTable.** Hand Calculation of Readability Scores
eReferences

Supplement 2.
Data Sharing Statement.
