# Supplementary material for: Comparison of Readability Scores for Written Health Information Across Formulas Using Automated vs Manual Measures
Source: JAMA Netw Open. 2022 Dec 12;5(12):e2246051. doi: 10.1001/jamanetworkopen.2022.46051 (PMC9856555; doi:10.1001/jamanetworkopen.2022.46051)
Supplement: Supplement 1. — eMethods. Detailed Methods eTable. Hand Calculation of Readability Scores eReferences [file jamanetwopen-e2246051-s001.pdf]

Supplementary Online Content

Mac O, Ayre J, Bell K, McCaffery K, Muscat DM. Comparison of readability scores for written health information across formulas using automated vs manual measures. *JAMA Netw Open*. 2022;5(12):e2246051. doi:10.1001/jamanetworkopen.2022.46051

eMethods. Detailed Methods

eTable. Hand Calculation of Readability Scores

eReferences

This supplementary material has been provided by the authors to give readers additional information about their work.

© 2022 Mac OA et al. *JAMA Network Open.*

eMethods. Detailed Methods

Selection of online readability calculators

We conducted a literature search on PubMed of studies assessing the accessibility of written health information that were published in 2021 to identify the most frequently used online readability calculators. We used the key word *Readability* alone or in combination with the following key words: *patient education material, online health information, assessment* and *evaluation*. Studies were excluded after title/abstract screening if they were published in a language other than English, publication was not in 2021, if they were a non-health topic, or if they did not include an objective assessment of readability. Studies were excluded after full-text screening if they did not use an online tool to calculate readability, did not report the online calculator used, or did not use one of the three included formulas (Simple Measure of Gobbledygook (SMOG) Index[1], Flesch Kincaid Grade Level (FKGL)[2] or Automated Readability Index (ARI)[3]). Studies were selected if they had been used in at least five studies and applied the formula correctly during the screening process. We also included two online calculators that are widely used in Australia[4,5], the Hemingway App[6] and the SHeLL Health Literacy Editor[7], which was developed by our team and is the only readability tool developed specifically for health information.

Screening of online calculators.

All calculators were screened for correct application of the readability formulas. This was to ensure that the formulas being applied incorrectly was not contributing to the variability in readability scores across online calculators. This involved comparing hand calculated scores with online readability scores of a short sample of text that contained only complete sentences (i.e., no headings or bullet points) and no numerals, acronyms or hyphenated words. A readability score within one grade reading level of the hand calculation was considered to have applied the formula correctly.

Text preparation

To assess the impact of text preparation, we followed existing guidelines for conducting readability analyses from the Centers for Medicare and Medicaid Services (CMS)[8]. This involved removing all incomplete sentences, adding full stops to any headings and bullet points or stems that were complete sentences, replaced any URLs with the word 'website' and removed phone numbers and mid-sentence periods (e.g., U.S).

Calculation of the reference standard

We calculated the SMOG Index, FKGL and ARI by hand to provide a reference standard to determine the accuracy of online readability scores. Readability scores were calculated independently by two researchers (OM and BC; eTable 1). Hand calculated scores were only performed on the prepared samples in line with recommendations to only include complete sentences in readability calculations[8]

.

© 2022 Mac OA et al. *JAMA Network Open.*

eTable. Hand calculation of readability scores

Readability formula	Hand calculation
SMOG Index	We counted the total number of words with three or more syllables and the number of complete sentences. All numerals and acronyms were fully syllabized (e.g., CDC was counted as three syllables and COVID was counted as two syllables). We then applied the following formula: $1.0430\sqrt{\text{number of words with 3 or more syllables } x \frac{30}{\text{number of sentences}}} + 3.1291$
FKGL	We counted total number of syllables, words and complete sentences. All numerals and acronyms were fully syllabized. Hyphenated words were counted as a single word. We then applied the following formula: $0.39\left(\frac{\text{total words}}{\text{total sentences}}\right) + 11.8\left(\frac{\text{total syllables}}{\text{total words}}\right) - 15.59$ Where there were discrepancies between the two researchers' calculations, we calculated the average of both scores.
ARI	We obtained the character count without spaces from Microsoft Word and validated it with an online calculator (Syllable Count)[9]. We then applied the following formula: $4.71\left(\frac{\text{characters}}{\text{words}}\right) + 0.5\left(\frac{\text{words}}{\text{sentences}}\right) - 21.43$

eReferences

1. McLaughlin GH. SMOG grading: A new readability formula. Journal of reading. 1969;12(8):639–646.
2. Flesch R. A new readability yardstick. Journal of Applied Psychology. 1948;32(3):221–33.
3. Smith EA, Senter RJ. Automated readability index. AMRL-TR Aerospace Medical Research Laboratories (6570th). 1967;1–14.
4. NPS MedicineWise. National Medicines Symposium Toolkit [Internet]. Sydney; 2022. Available from: https://www.nps.org.au/assets/NPSMW2458_NMS-2022-Toolkit_v2.pdf
5. Tasmanian Government Department of Health. Assessing Readability. 2019.
6. Long A, Long B. Hemingway Editor. 2013.
7. Ayre J, Muscat D, Bonner C, Mouwad D, Dalmazzo J, Harrison E, et al. Sydney Health Literacy Lab (SHLL) Health Literacy Editor. Sydney; 2021.
8. U.S. Department of Health and Human Services - Centers for Medicare & Medicaid Services (CMS). Using readability formulas: A cautionary note. Toolkit for making written material clear and effective [Internet]. 2010;1–39. Available from: https://www.cms.gov/Outreach-and-Education/Outreach/WrittenMaterialsToolkit/index.html
9. Arczis Web Technologies. Syllable Count [Internet]. Available from: https://www.syllablecount.com/

© 2022 Mac OA et al. *JAMA Network Open.*
