# Supplementary material for: Comparison of Readability Scores for Written Health Information Across Formulas Using Automated vs Manual Measures
Source: JAMA Netw Open. 2022 Dec 12;5(12):e2246051. doi: 10.1001/jamanetworkopen.2022.46051 (PMC9856555; doi:10.1001/jamanetworkopen.2022.46051)
Supplement: Supplement 2. — Data Sharing Statement. [file jamanetwopen-e2246051-s002.pdf]

Data Sharing Statement

Mac. Comparison of Readability Scores for Written Health Information Across Formulas Using Automated vs Manual Measures. *JAMA Netw Open*. Published December 12, 2022. doi:10.1001/jamanetworkopen.2022.46051

Data
Data available: No

Additional Information
Explanation for why data not available: Data can be made available upon reasonable request to the first author
